# Fabrication of UV-Stable Perovskite Solar Cells with Compact Fe_2_O_3_ Electron Transport Layer by FeCl_3_ Solution and Fe_3_O_4_ Nanoparticles

**DOI:** 10.3390/nano12244415

**Published:** 2022-12-10

**Authors:** Bangkai Gu, Yi Du, Song Fang, Xi Chen, Xiabing Li, Qingyu Xu, Hao Lu

**Affiliations:** 1 School of Physics, Southeast University, Nanjing 211189, China; 2 School of Materials Science and Engineering, Suzhou University of Science and Technology, Suzhou 215009, China

**Keywords:** perovskite solar cell, UV-stable, Fe_2_O_3_ film, electron transport layer, nanoparticles

## Abstract

Even though Fe_2_O_3_ is reported as the electron-transporting layer (ETL) in perovskite solar cells (PSCs), its fabrication and defects limit its performance. Herein, we report a Fe_2_O_3_ ETL prepared from FeCl_3_ solution with a dopant Fe_3_O_4_ nanoparticle modification. It is found that the mixed solution can reduce the defects and enhance the performance of Fe_2_O_3_ ETL, contributing to improved electron transfer and suppressed charge recombination. Consequently, the best efficiency is improved by more than 118% for the optimized device. The stability efficiency of the Fe_2_O_3_-ETL-based device is nearly 200% higher than that of the TiO_2_-ETL-based device after 7 days measurement under a 300 W Xe lamp. This work provides a facile method to fabricate environmentally friendly, high-quality Fe_2_O_3_ ETL for perovskite photovoltaic devices and provides a guide for defect passivation research.

## 1. Introduction

Organic–inorganic hybrid lead halide perovskites have attracted extensive attention [1,2]. Since the first reports in 2009, the power conversion efficiency (PCE) of PSCs has been improved to 25.7% within about one decade [3,4,5]. A typical planar PSC is composed of the structure of a cathode layer [6]. The ETL plays a significant role in electron extraction and transport from the perovskite absorber to the FTO [7]. To obtain highly efficient perovskite solar cells, a thin, transparent, and electrically conductive ETL without pinholes is crucial.

Currently, the most commonly used ETL material in PSCs is TiO_2_, owing to its high chemical stability, innate transparency, inexpensiveness, and appropriate conduction band (CB) level aligning with the perovskite layer [8]. However, TiO_2_-based devices are reported to suffer from hysteresis and high charge recombination, which severely restricts the wide use of the TiO_2_ ETL and hinders the development of PSCs [9]. Moreover, the photocatalytic properties of TiO_2_ could reduce the illumination stability of PSCs, resulting in poor UV light stability in PSCs [10]. Thus, a great deal of effort has been made to alleviate this problem. Meanwhile, many endeavors have been directed at searching for alternative semiconductor materials for ETLs, such as SnO_2_, ZnO, and Nb_2_O_5_ [11].

As an n-type semiconductor, iron oxide (Fe_2_O_3_) has attracted increased attention in photovoltaic applications, due to its high chemical stability, low cost, and suitable energy band position [12]. Considering its ultraviolet stability and visible light absorption, Fe_2_O_3_ is one of the most promising candidates for the ETL in PSCs. However, only several studies have been reported on the application of Fe_2_O_3_ in PSCs [13,14,15,16]. Wang et al. applied spin-coated Fe_2_O_3_ as the ETL in PSCs, attaining a PCE of 10.7%, with stability over 30 days upon exposure to ambient air, indicating high stability but a poor efficiency [14]. Guo et al. reported the application of Ni-doped Fe_2_O_3_ ETL, achieving an efficiency of 14.2%. They also reported the application of γ-Fe_2_O_3_ ETL fabricated at room temperature. However, it is difficult to fabricate Fe_2_O_3_ films with good conductivity and crystallinity [15,16].

Herein, we report an Fe_2_O_3_ ETL fabricated with the water-dispersed Fe_3_O_4_ nanoparticles and FeCl_3_ solution. It is found that the addition of FeCl_3_ in Fe_3_O_4_ nanoparticles precursor reduces the defects and enhances the passivation ability. As a result, the improved electron transfer and suppressed charge recombination contribute to an improvement in the short circuit current density (*J_sc_*) and open-circuit voltages (*V_oc_*), eventually yielding a champion PCE of 12.61%.

## 2. Experimental Section

### 2.1. Preparation of Fe_2_O_3_ ETLs

The ITO substrates were rinsed by ultrasonic vibration with acetone, ethanol, and deionized water for 30 min, and then treated with UV–ozone irradiation for 15 min.

A total of 600 mg of 2.2 mM FeCl_3_·6H_2_O (Alfa Aesar, 97%) and 300 mg of 1.5 mM FeCl_2_·4H_2_O (Alfa Aesar, 99%) was dissolved in 5 mL deionized water. Next, 800 mg of polyglucose sorbitol carboxymethylether was dissolved in 10 mL deionized water. Then, both of the solutions were mixed in a three-neck bottle, and stirred vigorously (300 rpm) with nitrogen gas bubbling. Then, the bottle was immediately transferred to a water bath at 60 °C, and 900 μL of 28% ammonium aqueous solution was added (stirring at 800 rpm). The bottle was transferred to a cryogenic bath (containing cold water, ice water, and ethanol). After cooling to −5 °C (decline rate 0.28 °C min^−1^), Fe_3_O_4_ nanoparticles solution was eventually obtained after workup by dialysis and filtration.

The Fe_2_O_3_ films fabricated with Fe_3_O_4_ nanoparticles were deposited on the substrates by spin-coating water-dispersed ten-nm-sized Fe_3_O_4_ nanoparticles with a concentration of 0.075 M at 5000 rpm for 30 s. The as-prepared layers were then annealed at 550 °C for 120 min in air. For the Fe_2_O_3_ films fabricated with FeCl_3_ solution, the precursor solution was prepared by dissolving FeCl_3_·6H_2_O (Alfa Aesar, 97%) in deionized water with a concentration of 0.075 M. The Fe_2_O_3_ films were deposited by spin-coating the prepared precursor solution at 4000 rpm for 30 s and sintered at 550 °C for 120 min in air. For the Fe_2_O_3_ films fabricated with FeCl_3_/Fe_3_O_4_ mixed solution, the mixed solution was prepared by dissolving FeCl_3_·6H_2_O in the as-prepared Fe_3_O_4_ solution with a concentration of 0.075 M. The Fe_2_O_3_ films were fabricated by spin-coating the mixed solution at 5000 rpm for 30 s, and then annealed at 550 °C for 120 min in air.

### 2.2. Fabrication of Perovskite Solar Cells

Perovskite solar cells were fabricated by a modified two-step method. Firstly, a PbI_2_ solution with 600 mg mL^−1^ in DMF was dropped on the ETL substrate with 3000 rpm for 30 s. A total of 50 µL of mixed solution (60 mg mL^−1^ FAI, 6 mg mL^−1^, MABr, and 6 mg mL^−1^ MACl in isopropanol) was then rapidly dripped on the rotating substrate 10 s after the spin procedure started. The as-prepared film was heated at 150 °C for 10 min in air in order to obtain a dense perovskite film. After cooling to room temperature, the HTL solution (spiro-OMeTAD, 25 µL) was deposited by spin-coating at 2000 rpm for 30 s. The HTL solution consisted of 72.3 mg spiro-OMeTAD, 28.8 µL 4-tert-butylpyridine (TBP), and 17.5 µL of 520 mg mL^−1^ lithium bis(trifluoromethylsulphonyl)imide (LiTFSI) in acetonitrile dissolved in 1 mL of chlorobenzene. Then, devices were oxidized in air for 36 h.

### 2.3. Characterization and Measurement

The surface morphology and cross-section of the samples were observed by a field-emission scanning electron microscope (FE-SEM, Hitachi, SU8010, Japan). The XRD results were measured with an X-ray diffractometer (XRD, Bruker, D8 Advance, Germany). The samples were also investigated by X-ray photoelectron spectroscopy (Thermo, Escalab 250Xi, USA). The photoluminescence (PL) and time-resolved photoluminescence (TRPL) were detected with a 530 nm laser (Edinburgh Instruments, LP320, UK). The absorption spectra were recorded on a UV–vis spectrophotometer (Shimadzu, UV-2600, Japan). The contact angle measurement was measured by DSA25E (KRÜSS, Germany). The current–voltage characteristics of the solar cells were tested with a Newport solar simulator and a Keithley 2400 Source Meter under AM 1.5G irradiation (100 mW cm^−2^). The electrochemical impedance spectroscopy (EIS) was measured with an electrochemical workstation (Autolab, PGSTAT 302 N, Switzerland) under AM 1.5G light condition with an alternative signal amplitude of 10 mV and in the frequency range of 0.1 Hz-40 kHz in glove box.

## 3. Results and Discussion

**Figure 1:**
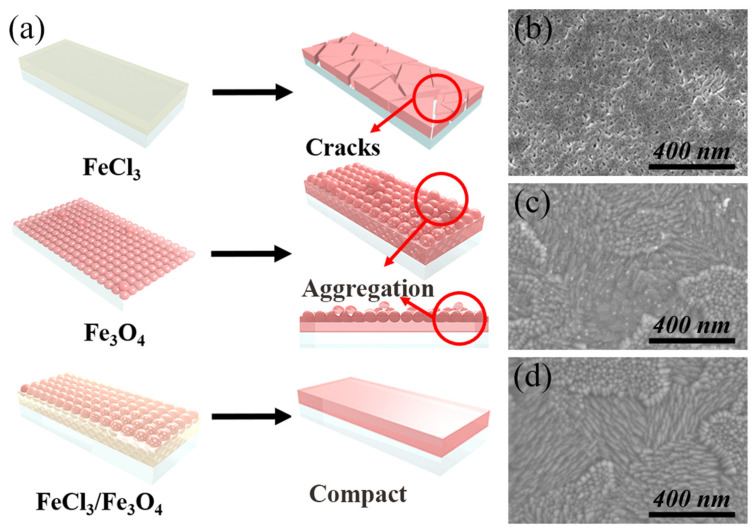
(**a**) Schematic of Fe_2_O_3_ films prepared by FeCl_3_ solution, Fe_3_O_4_ nanoparticles, and FeCl_3_/Fe_3_O_4_ mixed solution. Top-view SEM images of Fe_2_O_3_ film prepared by (**b**) 0.075 M FeCl_3_ solution, (**c**) water-dispersed ten-nm-sized Fe_3_O_4_ nanoparticles, and (**d**) FeCl_3_/Fe_3_O_4_ mixed solution.

Figure 1a exhibits the schematic of different Fe_2_O_3_ films prepared by FeCl_3_ solution, Fe_3_O_4_ nanoparticles, and FeCl_3_/Fe_3_O_4_ mixed solution. Fe_2_O_3_ films prepared by FeCl_3_ solution exhibit good compactness, but a large number of cracks and pin-holes after the annealing process. Fe_2_O_3_ films fabricated by water-dispersed Fe_3_O_4_ nanoparticles show better morphology. However, some aggregation is still found, owing to the gathered nanoparticles in the crystallization process, which could influence the nucleation process of perovskite film and suppress the charge transport at the Fe_2_O_3_/perovskite interface.

In order to further improve the planarity and compactness of Fe_2_O_3_ films, FeCl_3_ solution was incorporated into the Fe_3_O_4_ nanoparticle precursor solution, which could simultaneously retain the advantages of the two methods and reduce the defects, thereby facilitating an efficient ETL. Figure S1 shows the top-view scanning electron microscopy (SEM) image of blank and clean ITO substrate, as previously reported. As shown in Figure 1b, the Fe_2_O_3_ film prepared by 0.075 M FeCl_3_ solution shows a morphology with cracks and pin-holes, which could lead to direct contact between the perovskite absorber and ITO, resulting in aggravated charge recombination. Figure 1c shows the morphology of the Fe_2_O_3_ film fabricated by spin-coating water-dispersed ten-nm-sized Fe_3_O_4_ nanoparticles with a concentration of 6 mg mL^−1^ (measured by Fe), which demonstrates a flat and compact surface except for a few gathered spots. Figure 1d depicts the morphology of the Fe_2_O_3_ film prepared by FeCl_3_/Fe_3_O_4_ mixed solution. It can be observed that the as-prepared Fe_2_O_3_ film exhibits a pin-hole-free coverage, as a result of the cooperation between the nanoparticles and FeCl_3_ solution in the annealing process.

**Figure 2:**
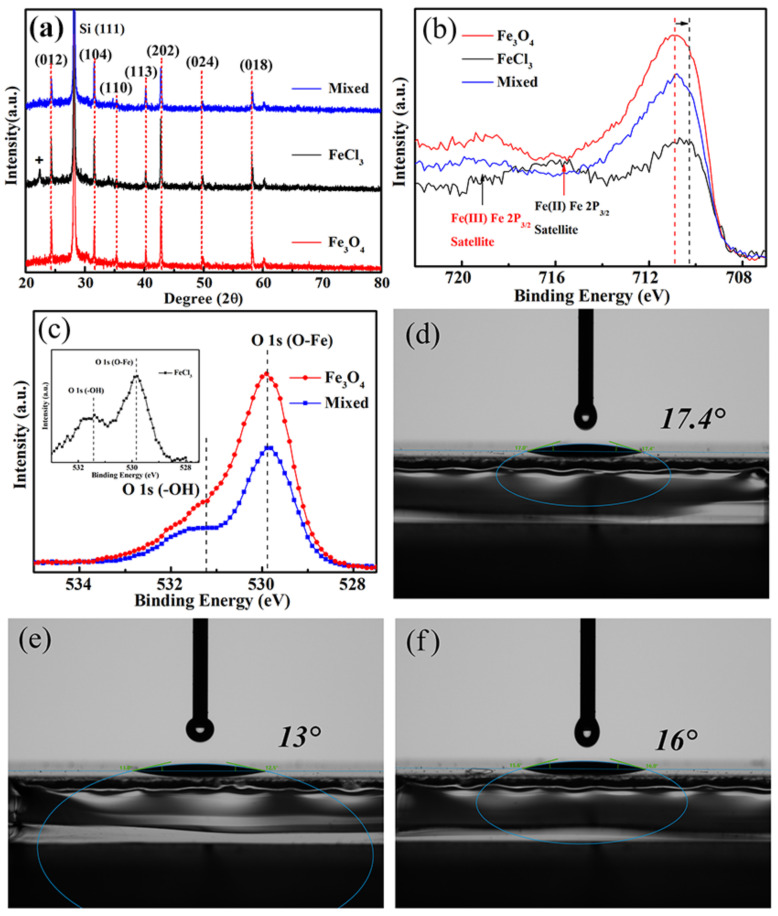
(**a**) XRD patterns, XPS spectra of (**b**) Fe 2P_3/2_ and (**c**) O 1s of Fe_2_O_3_ films prepared by FeCl_3_ solution, Fe_3_O_4_ nanoparticles, and FeCl_3_/Fe_3_O_4_ mixed solution, contact angle of Fe_2_O_3_ films prepared by (**d**) Fe_3_O_4_ nanoparticles, (**e**) FeCl_3_ solution, and (**f**) FeCl_3_/Fe_3_O_4_ mixed solution.

Figure 2a shows the X-ray diffraction (XRD) pattern of the Fe_2_O_3_ films prepared by different methods. XRD analysis confirms that both the samples prepared by Fe_3_O_4_ nanoparticles and FeCl_3_/Fe_3_O_4_ mixed solution display the same diffraction peaks, which match the standard α-Fe_2_O_3_ perfectly (JCPDS, No. 80-2377) [17]. XRD peaks at 22.5 and 24 degree may be the peaks of iron chlorate formed by the incompletely volatilized Cl in the crystallization process and the reduced iron. While the sample prepared by FeCl_3_ solution displays an extra peak at low angle. The XRD results indicate that Fe_3_O_4_ is converted into Fe_2_O_3_ and that the FeCl_3_/Fe_3_O_4_ mixed sample has better purity. X-ray photoelectron spectroscopy (XPS) measurements were carried out to elucidate the chemical composition of Fe_2_O_3_ films prepared by different methods.

Figure 2b illustrates the Fe 2p_3/2_ peak of the as-prepared Fe_2_O_3_ films. The fitted curves are shown in Figure S2. The peaks center around 716 eV and 719 eV, corresponding to the binding energy of Fe^2+^ ions and Fe^3+^ ions, respectively [18]. The curves of the samples prepared by Fe_3_O_4_ nanoparticles and FeCl_3_/Fe_3_O_4_ mixed solution show no obvious peak of Fe^2+^ ions, indicating that the Fe elements are converted into Fe_2_O_3_.

As shown in Figure 2c, a little peak of O-H can be observed in samples prepared by mixed solution, proving that there are intermediate products during the annealing process. To ascertain the influence of the different preparation methods on the surface energy, a contact angle test was carried out on the as-prepared Fe_2_O_3_ substrates. The contact angles are 13°, 17.4°, and 16° for Fe_2_O_3_ films prepared by FeCl_3_ solution, Fe_3_O_4_ nanoparticles, and FeCl_3_/Fe_3_O_4_ mixed solution, respectively. For the FeCl_3_ prepared sample, the smallest contact angle could arise from its terrible morphology with large-area cracks and pin-holes, which could trap the perovskite precursor solution. It should be noted that the FeCl_3_/Fe_3_O_4_ mixed sample has a smaller contact angle than that of Fe_3_O_4_ nanoparticles. Attributing this to the addition of FeCl_3_ solution, the defects and aggregation of the Fe_3_O_4_ prepared films are passivated, leading to a compact and flat coverage of the Fe_2_O_3_ film prepared by FeCl_3_/Fe_3_O_4_ mixed solution. The reduced defects and passivated surface of the Fe_2_O_3_ films make a great contribution to a smaller contact angle, which is conducive to the diffusion of perovskite precursor solution on the surface, thus, accelerating the nucleation process of perovskite films [19]. Figure S3 illustrates the UV–vis absorption spectra of Fe_2_O_3_ films prepared by different methods. The Fe_2_O_3_ film prepared by FeCl_3_/Fe_3_O_4_ mixed solution shows a slightly higher absorption in almost the whole wavelength region, which could prevent the perovskite from degrading under UV irradiation and enhance the UV-stable ability.

**Figure 3:**
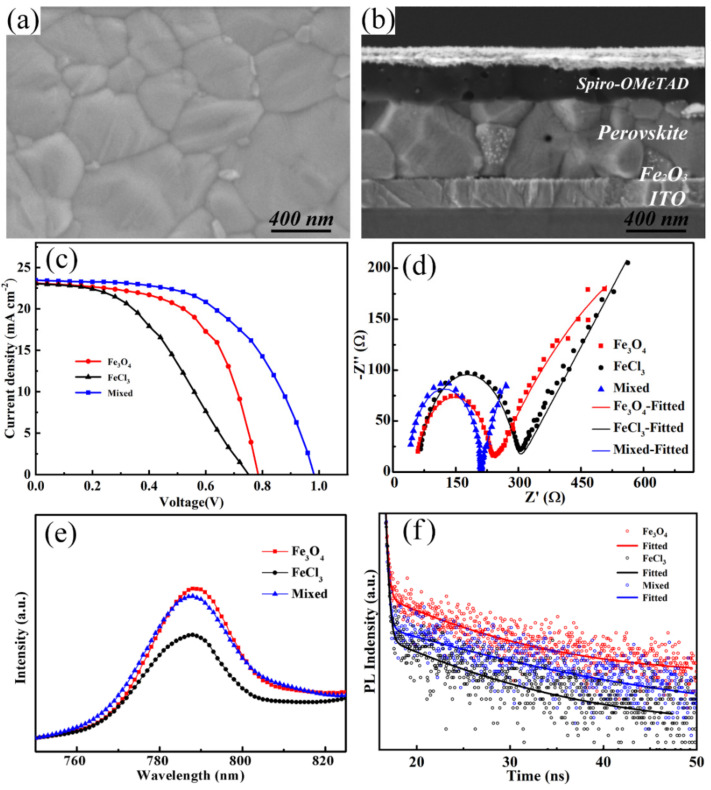
(**a**) Top-view SEM image of the perovskite layer. (**b**) Cross-sectional SEM image of the entire structure. (**c**) *J-V* curves of PSCs based on Fe_2_O_3_ ETLs prepared by different methods. (**d**) Nyquist plots of PSCs based on the Fe_2_O_3_ ETLs prepared by different methods. (**e**) PL spectra, (**f**) TRPL spectra of perovskite based on Fe_2_O_3_ films prepared by different methods.

Figure 3a shows the top-view SEM image of the perovskite layer deposited on the FeCl_3_/Fe_3_O_4_ mixed sample, which exhibits compact surface and large grain size. Figure 3b shows the cross-sectional SEM image of the entire structure, from which we can see the perovskite layer is also compact and the thickness is about 500 nm. Figure S4 shows the XRD patterns of perovskite coated on as-prepared substrates, and all the peaks of the perovskite are presented with an asterisk. All of them display the same characteristic peaks of perovskite materials, which indicates excellent perovskite crystallinity [20]. Figure 3c presents the best current density–voltage (*J-V*) curves of the devices based on Fe_2_O_3_ films prepared by different methods. All samples were measured under AM 1.5G (from 1.2 V to 0 V, scan step of 0.04 V, and scan rate of 100 mV s^−1^). The devices based on Fe_2_O_3_ films are also compared with the TiO_2_-based device, as shown in Figure S5. The detailed photovoltaic parameters of the PSCs with the best PCE values including open-circuit voltage (*Voc*), short-circuit current density (*J_SC_*), filling factor (*FF*), and PCE are summarized in Table S1. The device prepared with FeCl_3_ displays the lowest PCE of 7.72% and the device based on Fe_3_O_4_ nanoparticles provides a PCE of 10.64%. Expectedly, the optimal device prepared by mixed solution exhibits overall superior performance, including a *Voc* of 0.98 V, *Jsc* of 23.45 mA cm^−2^, and *FF* of 54.74%, resulting in a PCE of 12.61%. Compared with the device based on single Fe_3_O_4_ nanoparticles, *Voc* and *Jsc* are improved, which may be due to the reduced defects and passivated recombination with the addition of FeCl_3_. The forward and reverse scanning tests were also carried out to investigate the hysteresis effect by (PCE_reverse_ − PCE_forward_)/PCE_reverse_. As shown in Figure S6, the mixed sample shows a minimum hysteresis of 0.09. As a contrast, the FeCl_3_ prepared sample shows a hysteresis index of 0.15, and that of the Fe_3_O_4_ prepared sample is 0.10. It is indicated that PSC based on the mixed sample shows a better charge-transfer ability. Further characterizations were performed to evaluate the trap state density of the devices. We prepared electron-only devices with structures of ITO/ETL/perovskite/PCBM/Ag to quantitatively assess the trap state density in ETL, as shown in Figure S7. Compared with the Fe_3_O_4_-based device, the V_TFL_ of the mixed sample is reduced to 0.12 V. It is indicated that that addition of FeCl_3_ can obtain high-quality Fe_2_O_3_ film with compact and flat coverage, contributing to passivating the surface defect and effectively filling the electron trap density, which can greatly improve the electrical properties and accelerate electron extraction and injection at the ETL/perovskite interface.

To investigate charge transport and recombination in perovskite solar cells, electrochemical impedance spectroscopy (EIS) was conducted. Figure 3d shows the Nyquist plots of the devices based on Fe_3_O_3_ ETLs prepared by different methods under AM 1.5 G illumination, and the fitted parameters are summarized in Table S2. The semicircle at high frequency is related to the transfer resistance (*R_ct_*) at the interface and the semicircle at low frequency corresponds to recombination impedance (*R_rec_*) of the device [21]. The device based on FeCl_3_/Fe_3_O_4_ film exhibits a *R_ct_* of 178 Ω and *R_rec_* of 1023 Ω. The reduced *R_ct_* is conducive to the enhanced the carriers transfer at the interface, and the increased *R_rec_* is beneficial to the suppressed charge recombination. To further investigate the leakage capacity of Fe_3_O_3_ ETLs prepared by different methods, a leakage current test is carried out, as shown in Figure S8. The FeCl_3_/Fe_3_O_4_-based sample shows the lowest leakage value, indicating a better leakage performance.

Photoluminescence (PL) was carried out to explore the carrier transport dynamics at the Fe_2_O_3_/perovskite interface, as shown in Figure 3e. All the samples display a typical emission peak at 788 nm, in agreement with the absorbance edge of the perovskite. The FeCl_3_ prepared sample presents the lowest PL intensity. This could mainly be correlated to poor coverage of the prepared film, which could cause the direct contact between perovskite and ITO, resulting in an illusion of great electron transfer and extraction. A higher PL intensity is presented in the sample with Fe_3_O_4_-prepared films. It should be ascribed to the imperfect surface and interface. The FeCl_3_/Fe_3_O_4-_prepared film demonstrates a PL quenching, indicating that the addition of FeCl_3_ can passivate the surface defect and accelerate electron extraction and injection at the ETL/perovskite interface. To further demonstrate the charge transfer and extraction, the time-resolved photoluminescence (TRPL) was performed. Figure 3f shows the TRPL spectra and the fitting curves with a bi-exponential decay function [22]. It is clear that the average recombination lifetime (*τ*_ave_) is prolonged from 7.76 ns to 20.28, and 12.29 ns for samples with FeCl_3_, Fe_3_O_4,_ and FeCl_3_/Fe_3_O_4-_prepared films, respectively. Compared to the Fe_3_O_4-_ prepared sample, the decreased carrier lifetime of the FeCl_3_/Fe_3_O_4-_prepared sample indicates that the addition of FeCl_3_ passivates defects of the Fe_3_O_4_ prepared films and greatly accelerates the charge separation and transport, leading to suppressed charge recombination.

**Figure 4:**
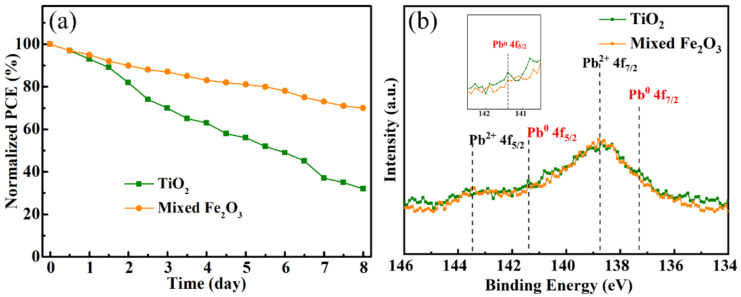
(**a**) Long-time stability test under AM 1.5 G of controlled TiO_2_ and mixed-SnO_2-_ETL-based perovskite solar cells. (**b**) XPS spectra depicting Pb 4f_5/2_ and Pb 4f_7/2_ peaks of controlled TiO_2_ and mixed-SnO_2-_ETL-based perovskite solar cell, which were tested for long-time stability for 7 days.

The transmittance spectra of Fe_2_O_3_ films prepared by different methods are shown in Figure S9. The FeCl_3_/Fe_3_O_4_-prepared film shows a high transmission, but it is still slightly lower than that of the TiO_2_ film. Figure 4a shows the long-time stability test of controlled TiO_2_ and mixed Fe_2_O_3-_ETL-based perovskite solar cell, which were tested under a 300 W Xe lamp with the condition of humidity of less than 20% and temperature of 25 ℃. In order to obtain more accurate stability test results, we used Au as the top electrode instead of the original Ag. The efficiency of the device prepared by controlled TiO_2_ ETL decreases more than 70% after 7 days of continuous irradiation. As the most commonly used ETL material in PSCs, TiO_2_ is reported as a serious issue that affects the stability of the PSCs. As a product of the TiO_2_ photocatalytic effect, UV illumination can excite TiO_2_ to generate strong oxidizing holes, which could cause the decomposition of perovskite into CH_3_NH_2_, HI, and PbI_2_, and eventually result in the degradation of the stability [23,24,25]. The device prepared with mixed Fe_2_O_3_ ETL still has 70% efficiency, indicating a better stability performance. We speculate that it is due to the UV stability and lesser photocatalytic ability of Fe_2_O_3_, which slows the perovskite from degradation and, thus, enhances the UV-stable ability of PSCs. We also tested the TiO_2_ and mixed-Fe_2_O_3_-based devices at the maximum power point (MPP) to investigate the stability under UV illumination (composed of 313 nm, 340 nm, and 351 nm) without encapsulation, as shown in Figure S10. Under the same conditions for 300 min, the mixed-Fe_2_O_3_-based device retains 86% of its initial current density, while the current density of the TiO_2_-based device only retains 52%, indicating no UV reaction of Fe_2_O_3_ and perovskite, which makes a great contribution to the UV-stable devices. To further confirm our point of view, the XPS measurements were carried out to elucidate the valance change of Pb in the perovskite of controlled TiO_2_ and mixed-Fe_2_O_3-_ETL-based perovskite solar cells, which were tested for long-time stability for 7 days. Figure 4b shows the peak of the XPS spectra centered at 141.4 eV, corresponding to Pb^0^ 4f_5/2_ of controlled TiO_2-_based sample, which is in good agreement with the literature values of 141.7 eV [26]. The peak is higher than that of the Fe_2_O_3_-based sample, confirming the presence of unsaturated Pb, which results from the degradation of perovskite and could be detrimental to the instability of the sample. For the Fe_2_O_3_-based sample, the peak of Pb^0^ 4f_5/2_ is successfully suppressed, indicating improved stability of the perovskite. We think that the improvement of the stability should be ascribed to no UV reaction of Fe_2_O_3_, which protects perovskite from degradation under continuous irradiation.

## 4. Conclusions

In summary, we present a facile modification with FeCl_3_ solution to optimize the Fe_2_O_3_ ETL prepared by water-dispersed Fe_3_O_4_ nanoparticles. The device efficiency is improved by more than 118% for the optimized device. The stability efficiency of the Fe_2_O_3_-ETL-based device is nearly 200% higher than that of the TiO_2_-ETL-based device after 7 days measurement. The improved performance of the as-prepared solar cells is attributed to the reduced defects at the interface, enhanced passivation ability, excellent perovskite crystallization originating from the addition of the FeCl_3,_ and the UV-stable ability of the Fe_2_O_3_-based devices. This work is dedicated to broadening the scope of perovskite photovoltaic devices and provides a way for defect passivation in commercial applications.

## Data Availability

The data is available on reasonable request from the corresponding author.

## Supplementary Materials

The following supporting information can be downloaded at: https://www.mdpi.com/article/10.3390/nano12244415/s1, Figure S1. Top-view SEM images of pure ITO; Figure S2. Fitted curves of the Fe 2P_3/2_ of Fe_2_O_3_ films prepared by different methods; Figure S3. UV–vis absorption spectra of Fe_2_O_3_ films prepared by different methods; Figure S4. XRD patterns of perovskite coated on different substrates; Figure S5. *J-V* curves of PSCs based on Fe_2_O_3_ and TiO_2_ ETLs; Figure S6. Hysteresis measurement of PSCs based on Fe_2_O_3_ prepared by different methods; Figure S7. Current−voltage curves of the PSCs with a structure of ITO/HTMs/perovskite/spiro-OMeTAD/Ag; Figure S8. Leakage current measurement of PSCs based on the Fe_2_O_3_ ETLs prepared by different methods; Figure S9. Transmittance spectra of TiO_2_ and Fe_2_O_3_ films prepared by different methods. Figure S10. The continuous illumination stability of the TiO_2_ and mixed Fe_2_O_3_ based devices under UV illumination without encapsulation; Table S1. Summary of photovoltaic parameters of the PSCs based on the control TiO_2_ ETLs and Fe_2_O_3_ ETLs prepared by different methods (30 devices tested in the reversed direction); Table S2. Summary of the fitted parameters of solar cells based on the Fe_2_O_3_ ETLs prepared by different methods.
