# Supplementary material for: Fabrication of UV-Stable Perovskite Solar Cells with Compact Fe2O3 Electron Transport Layer by FeCl3 Solution and Fe3O4 Nanoparticles
Source: Nanomaterials (Basel). 2022 Dec 10;12(24):4415. doi: 10.3390/nano12244415 (PMC9781711; doi:10.3390/nano12244415)
Supplement: Supplementary file 1 [file nanomaterials-12-04415-s001.zip › nanomaterials-2048673-supplementary.pdf]

Supplementary Materials

Fabrication of UV-Stable Perovskite Solar Cells with Compact Fe_2O_3 Electron Transport Layer by $FeCl_3$ Solution and Fe_3O_4 Nanoparticles

Bangkai Gu [1,2,†], Yi Du [2,†], Song Fang [2], Xi Chen [2], Xiabing Li [2], Qingyu Xu [1,*] and Hao Lu [2,*]

1 School of Physics, Southeast University, Nanjing 211189, China
2 School of Materials Science and Engineering, Suzhou University of Science and Technology, Suzhou 215009, China

* Correspondence: xuqingyu@seu.edu.cn (Q.X.); haolu@usts.edu.cn (H.L.)
† These authors contributed equally to this work.

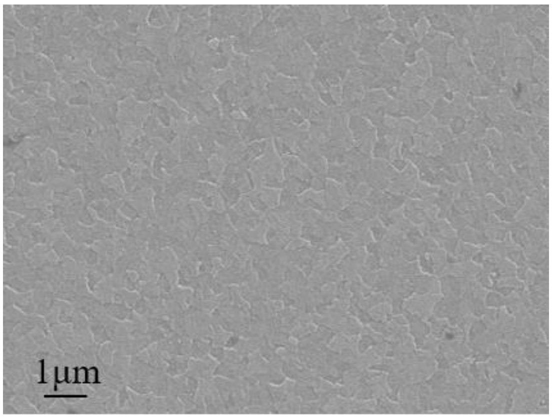

Figure S1. Top view SEM images of pure ITO.

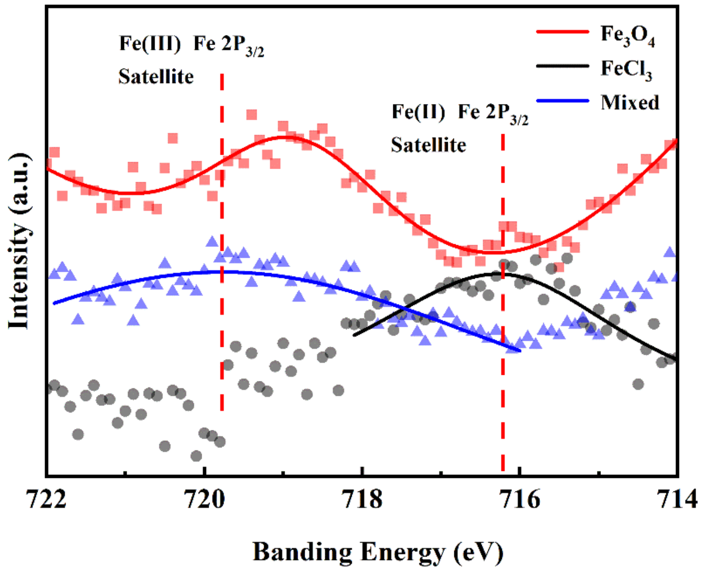

Figure S2. Fitted curves of the Fe $2P_{3/2}$ of Fe_2O_3 films prepared by different methods.

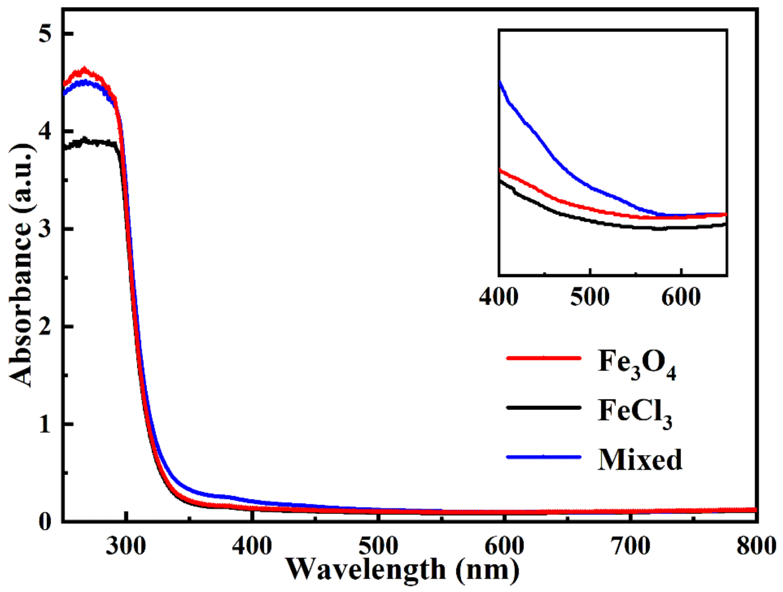

Figure S3. UV–vis absorption spectra of Fe_2O_3 films prepared by different methods.

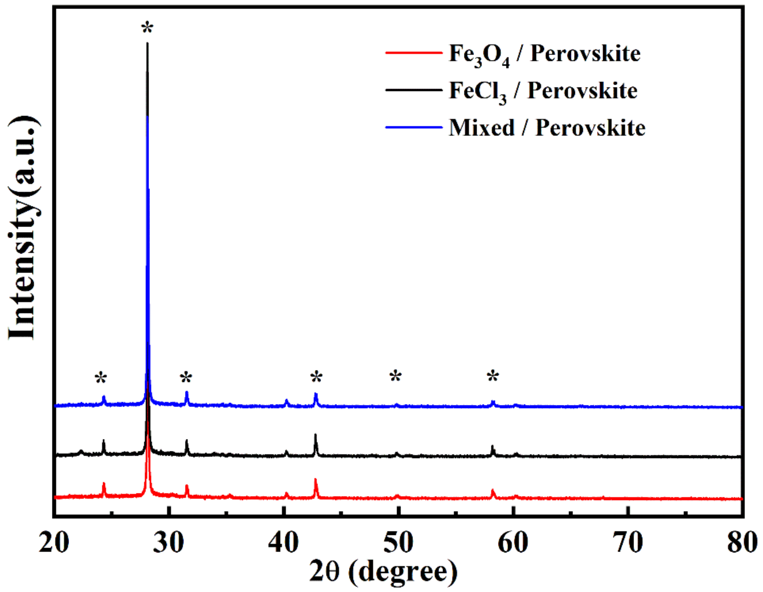

Figure S4. XRD patterns of perovskite coated on different substrates.

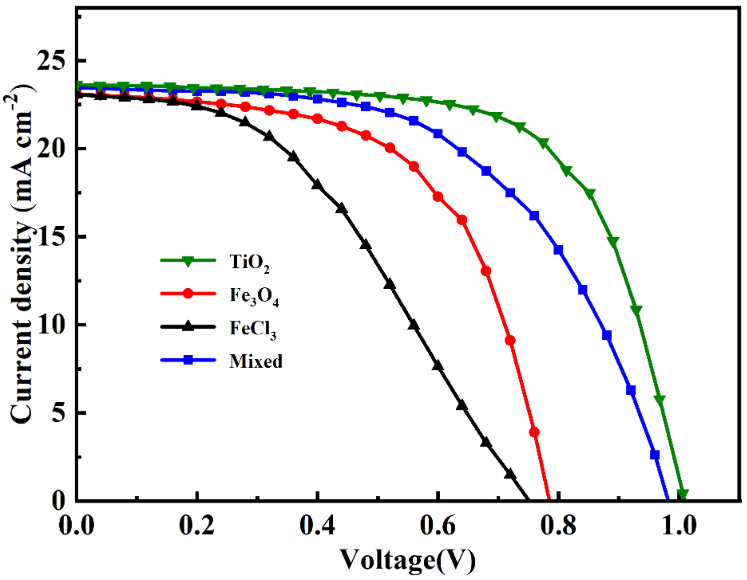

Figure S5. *J-V* curves of PSCs based on Fe_2O_3 and TiO_2 ETLs.

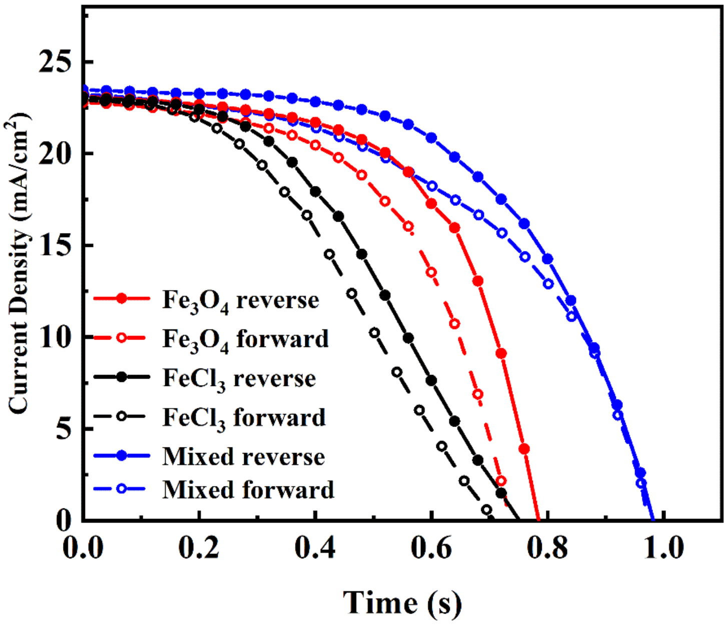

Figure S6. Hysteresis measurement of PSCs based on Fe_2O_3 prepared by different methods.

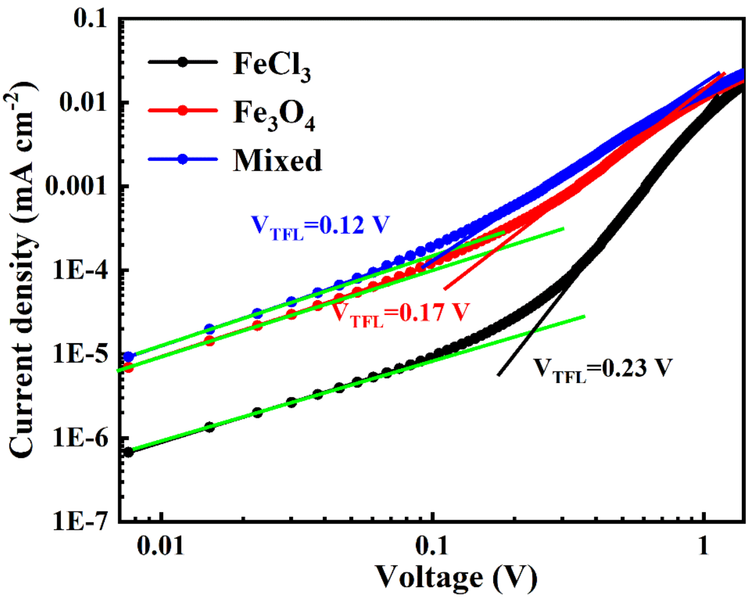

Figure S7. Current−voltage curves of the PSCs with a structure of ITO/HTMs/perovskite/spiro-OMeTAD/Ag.

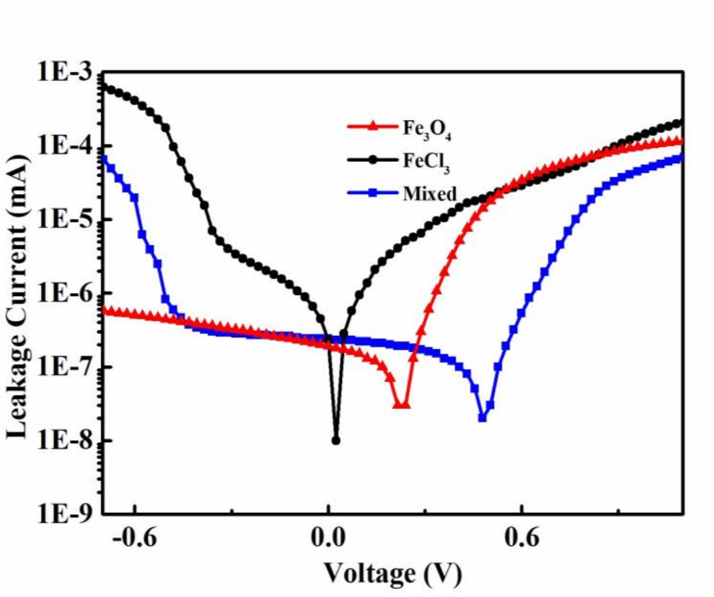

Figure S8. Leakage current measurement of PSCs based on the Fe_2O_3 ETLs prepared by different methods.

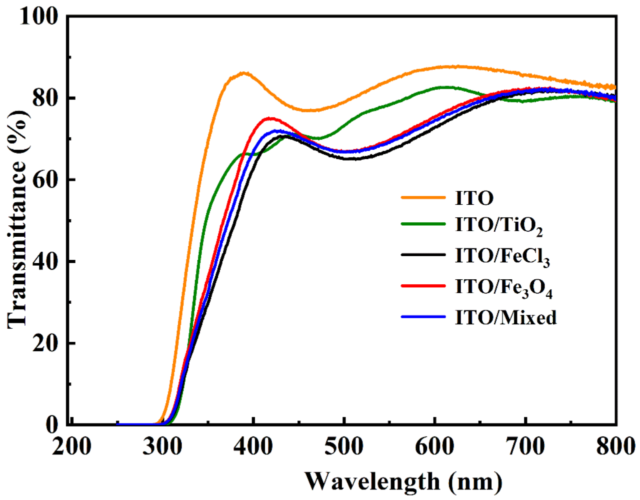

Figure S9. Transmittance spectra of TiO_2 and Fe_2O_3 films prepared by different methods.

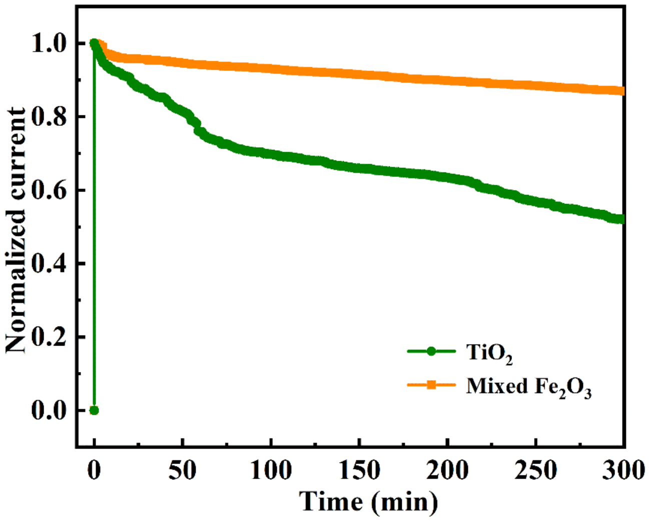

Figure S10. The continuous illumination stability of the TiO_2 and mixed Fe_2O_3 based devices under UV illumination without encapsulation.

Table S1. Summary of photovoltaic parameters of the PSCs based on the control TiO_2 ETLs and Fe_2O_3 ETLs prepared by different methods (30 Devices Tested in the Reversed Direction).

Devices		V_{oc} (V)	J_{sc} (mA cm^{-2})	*FF* (%)	*PCE* (%)
TiO_2	Best	1.01	23.64	66.17	15.50
	Average	0.99± 0.02	22.91± 0.73	64.85± 1.32	14.47± 1.03
Fe_3O_4	Best	0.78	23.12	58.63	10.64
	Average	0.74 ± 0.04	22.17 ± 0.95	55.58 ± 3.05	9.15 ± 1.49
$FeCl_3$	Best	0.77	23.10	43.52	7.72
	Average	0.73 ± 0.04	22.38 ± 0.72	41.57 ± 1.95	6.30 ± 1.42
Mixed	Best	0.98	23.45	54.74	12.61
	Average	0.95 ± 0.03	22.89 ± 0.56	52.66 ± 2.08	11.56 ±1.05

Table S2. Summary of the fitted parameters of solar cells based on the Fe_2O_3 ETLs prepared by different methods.

Devices	R_s (Ω)	R_{ct} (Ω)	R_{rec} (Ω)	τ (ns)
Fe_3O_4	58.2	270	573	20.28
$FeCl_3$	54.5	212	620	7.76
Mixed	25.4	178	1023	12.29
